# Memoires de l'Academie Royale de Médicine. Tome IX

**Published:** 1842-04-01

**Authors:** 


### Memoires de l'Academie Royale de Médecine. Tom. IX. pp. 796. Paris: Baillière, 1841.

This annual collection of the Memoirs which have been read before, and pronounced worthy of publication by, the Paris Academy of Medicine, much resembles the Medico-Chirurgical Transactions of our own country. The present volume is an interesting one, and we proceed to give an account of the various papers it contains, omitting, however, as foreign to our purpose, any notice of the *éloges* upon Tessier, Sanson, and Ambroise Paré.

### I. Report of the Committee upon the Epidemics which have prevailed in France during 1839-40. By *M. Bricheteau.*

Documents connected with this subject have been forwarded to the central authorities from the Prefects of most of the Departments.

1. *Typhoid Fevers.*—Of the communications relating to epidemics, amounting to above 80, thirty relate to severe epidemic *fevers*—the term typhoid being, as M. Bricheteau observes, most vague, embracing the forms of disease of various origin, and various nature. Typhoid fever is endemic in Britain, France, Germany, and Sweden, as is the plague in the East, the cholera in India, and the yellow fever in America—and, like all these diseases, may, at certain seasons become epidemic. Like these, also, it usually attacks but once the same individual, and its specific treatment has yet to be discovered. In the districts embraced by the reports there were 1,433 cases and 255 deaths. In six epidemics the *cause* was stated to be contagion, but the facts were too loosely stated: in two, over-crowded cemeteries, surrounded by habitations; in six, the vicinity of marshes and cloacæ, and general neglect of hygiene: in others, atmospheric vicissitudes, famine and poverty. The *symptoms* were frequently of a very grave character, those of the worst form of typhus, and were often attributed to the injudicious use of stimuli and purgatives, and to the intermeddling of the ignorant and quacks, before proper assistance could arrive. Even regular practitioners, says the report, do not seem to have had any defined notions upon the subject of treatment, merely attacking symptoms, and not pursuing any exclusive practice, whether tonic or antiphlogistic.

2. *Remittents and Pernicious Intermittents.*—When they are mild in their attacks, intermittents, so far from being dreaded in the localities wherein they appear, are frequently desired, being supposed to be preventives of more serious evils; but, when they prove malignant, they spread general alarm. Three such epidemics are here noted, and their examination proves that many localities are as yet absolute strangers to all hygienic precautions: large marshes, stagnant pools, cloacæ, &c. existed in the immediate vicinity. The affection usually manifested itself at first in a very obscure manner, and the mortality was very considerable, until *quinine* was given. Large quantities were required, sometimes 200 or 300 grains, before relief was obtained. Indeed, in such cases, quantity must never be regarded, for no ill effects upon the intestinal canal or spleen ever occurred from the use of this drug. In one of these epidemics, complicating pneumonia, the use of the lancet was found to be followed by alarming debility, and a cure rather to be obtained from anodynes, quinine, and counter-irration.

3. *Dysentery.*—This prevailed epidemically and endemically, in Brittany and La Vendée, caused by atmospheric vicissitudes and unwholesome diet. In these provinces it often puts on the form of cholera. At the commencement, the mortality was terrible—of six seized only one escaping. *Opium* proved the sheet-anchor. The rapidity of the course of the disease was remarkable. If the patient were not relieved, he sank in four or five days, but, in more favourable cases, eight days were occupied by the malady.

4. *Miliary Sweat.*—This epidemic disease, prevailing especially in Picardy, has been long known as the "Picardy Sweat." It first appeared in 1718. In 1733 it gave rise to Bellot's celebrated description. Attacks have since occurred from time to time; but the severest of all was the epidemic of 1821, so well described by Rayer. In the present report two epidemics are noted: in one, twenty persons died out of 77 attacked, and in the other, 16 out of 115: the one arose without appreciable cause; the other was thought to be due to the vicinity of a hemp-dresser, and an animal charcoal maker; in the one, early bleeding was very useful, but not so in the other.

Among the various reports on epidemics of scarlatina, small-pox, measles, &c. the committee only allude to one, containing a case of *rubeola nigra*, observed at Rheims.

It seems that there are medical men of note appointed in each district to repair, upon the appearance of epidemics, to the spot, superintend the treatment, and report upon the result. It is an excellent arrangement, seeing how badly some of the provincial districts of France are supplied with medical attendants; but, as the committee observes, these medical authorities, sometimes arriving only when the epidemic was upon its decline, have unwittingly deceived themselves as to the superior efficacy of the treatment they adopted—a remark we imagine that may justly be applied to similar hasty conclusions, which are sometimes drawn in our own country.

The committee dwells strongly upon the importance of improving the condition of the habitations of the lower classes. For want of attention to this, Normandy and Picardy, two of the finest provinces in France, are afflicted with epidemics. "Who would believe, that in the midst of a refreshing air, and of a vegetation which purifies the atmosphere, our peasants are living, during the long nights of Winter, nearly asphyxiated in their narrow cabins, where all ages are confounded together, and where they are sometimes disputing with their domestic animals a few cubic feet of respirable air? In such habitations, humidity, mephitic vapours, the exhalations from dung-heaps, and putrifying substances, and stagnant waters, favor the development of dreadful diseases, and ravaging epidemics."

The populous city and large town, with their cellars and garrets, can tell, we fear in our own country, even a yet more fearful tale of physical suffering and moral depravation. Public attention is however becoming aroused to the necessity of remedying this state of things, evidences of which we take to be the laying out of new parks, the widening of streets, the improvement of the drainage, and the contemplated discontinuance of interment in towns.

### II. A Memoir on several recent Trials for Poisoning by Arsenic. By M. *Orfila*.

Our readers are aware of the nature and importance of M. Orfila's recent improvements in the mode of detecting arsenic.* The early disappearance of the poison from the alimentary canal heretofore rendered its detection, when existing in small quantities, impossible after a brief period. M. Orfila has found that it is to be discovered at a later period in the urine, with which it has been in part eliminated from the system, and still later in the substance of various organs, by destroying the animal matters of portions of these, and then submitting the residue to the action of Marsh's apparatus. As the portion so obtained is frequently very minute, considerable delicacy and adroitness are required in conducting the investigation; and it becomes of great importance, that the various proofs offered by this distinguished chemist, that the spots resulting from the action of Marsh's apparatus are really arsenical, be submitted to the most rigid investigation. Many cases, hitherto supposed to be beyond the pale of justice, will, if M. Orfila's researches be confirmed, be brought under its cognizance; and no name, however great, can render diversified and repeated experiment unnecessary, when so many lives may eventually depend upon the establishment of this new test. It must ever be remembered that tests heretofore vaunted as certain, and upon the faith of which condemnations or acquittals have taken place, by improvements in science have been proved fallacious. However, into this investigation we have now no purpose of entering, but wish simply to notice some examples of the practical employment of the new test in France. M. Orfila reports five cases which have been recently decided by the tribunals.

In the *first*, a man was supposed, from the symptoms during life, and the appearances after death, to have been poisoned by his wife. Nine months after his death, decoctions of various parts of his body were sent to M. Orfila, and, he finding no trace of arsenic, the woman was acquitted.

The *second* case was that of a youth, supposed to have been poisoned by his father. His body was not examined until fourteen days after death, when the mucous membrane of the stomach and duodenum were found ulcerated in places. No traces of poison were discovered in the analysis

* Medico-Chir. Review. Nos. 63, 64, 66 and 67.

of the contents of the stomach. Three months after, the body was disinterred, placed in a new barrel, and sent to Orfila. It was in pieces, some of the organs being decomposed, and exhaled a horrible odour. An extract of various portions of the body, having been produced by long decoction, the animal matter destroyed, and the residue placed in Marsh's apparatus, the arsenical spots appeared. The body of a dog, which had been poisoned by arsenic, yielded the same appearances, while, these were absent in two bodies in whom no suspicion of poison existed, and which were submitted to the same processes. The earth of the cemetery was examined in several samples, but only in one instance did slight traces of arsenic present themselves. The accused was accordingly condemned. The trial was remarkable, as M. Raspail presented himself in order to combat Orfila's opinions. We have already said, that the new views of this latter chemist must be well examined into, and, if found erroneous, they must of course be opposed; but such opposition to them must be conducted in the same spirit of fairness, and with the same desire for the advancement of chemical science, and medical jurisprudence, as have prevailed in their promulgation. Men of science will not consent to be represented by so fantastic and so prejudiced a person as M. Raspail has proved himself on many occasions to be. Admitted as an evidence for the accused, he entered upon a vague declamatory tirade, during which, personal enmity, and an ignorance of the matter in dispute, became too apparent. Thus, the phenomena of putrefaction, the enthusiasm for new experiments, and the abandonment of old tests were all cited. The failure of the other chemists to detect arsenic in the contents of the stomach, he held a sufficient proof that none existed. But, supposing that arsenic really had been detected in the liver, muscles, &c. of this person, might not the barrel have been infected, or may not the poison have been introduced surreptitiously during the exhumation? Is there not arsenic in vegetables, and will not paper coloured with arseniate of copper infect manure in contact with it? Why was only a killogramme of the earth of the cemetery examined? its examination should have been conducted on a grand scale. If, as is possible, arsenic should have been found in this earth, who can tell, but that by some unknown internal movements, or electrical agency, (!) it may not have reached the substance of the viscera of the corpse! The discoveries of no man, however eminent, must be taken as law, unless *academies* have decided in favour of them! Have not the most skilful chemists admitted that the decoction of onion will produce the same re-actions as arsenic? This apparatus of Marsh is liable to betray its employers into many errors.

The judge, pointing to some of the spots which had been produced by submitting the liver to the process, demanded of M. Raspail whether these were arsenical. They look like it, replied he, but there are a thousand substances which, if combined in this or that manner, would produce the same appearance. "Will you give us an example of some of these substances, Sir." "That would occupy too much time; such problems as these are not to be resolved in a few hours; besides I am not here to make experiments. But look at this apparatus of Marsh, every means is abandoned for it, because it is superior to all; and, yet, when by it the arsenic is obtained, to recognise its existence, you are obliged to have recourse to the very tests you condemn."

"If this vague theory respecting the arsenic of soils be true," replied M. Orfila, "there is an end of examining any bodies which have been once interred. Let bodies be disinterred from the cemeteries of the Bicetre, or Mont Parnasse, the soil of which is avowedly slightly arsenical, and I will defy you to discover in the organs of these bodies any traces of arsenic. The idea of a subterraneous electricity producing such extraordinary effects is purely imaginary. If I renounce all other means in favour of Marsh's apparatus, it is because this is the only one which will shew these small quantities of metallic arsenic; but, I do not deny the efficiency of other tests, when we have a sufficiency of the metal to operate upon. When by the aid of this apparatus we have obtained minute metallic atoms, which would have escaped us by any other proceeding, we are still obliged to prove that the metal so produced is really arsenic, by submitting it to the action of fire, nitric acid, nitrate of silver, &c. I exposed the errors respecting the decoction of onion twenty years ago. M. Raspail says these spots are not produced by arsenic. My reply is short. Let him cite any substance, uniting the characters I have detailed, and prove the exactitude of his assertion,—then, instantly, I will tear my report into pieces, and beg the jury to regard all I have said and written upon this subject as if it had never occurred."

In the *third* case, a woman perished with the symptoms of poisoning, and several lesions were found in the alimentary canal. Some chemists had tested the contents of the stomach, &c. and a portion of the organs were submitted to the processes recommended by Orfila. A decoction of the stomach gave very slight traces of arsenic; but these Orfila considered sufficient, as the woman having lived five days, there had been ample time for the greater portion of the poison to be discharged by vomiting, and eliminated by the urine. He repeated the experiment, and produced the spots which he considered conclusive. M. Raspail did not appear personally, but transmitted a pamphlet with new objections, and among other assertions respecting the detection of arsenic, he states that negative results are irrefragable proofs of non-poisoning in the case of the metallic poisons—thus, putting the relative skill of the operator, and excellence of his process, entirely out of the question.

The husband was condemned, and it was afterwards discovered that he had obtained arsenic. *The grocer who sold it him was fined* 3000 *francs* (£125.) *for so doing.*

During this trial, the judge asked M. Orfila, whether the fact of the vomiting continuing for five days, could be explained upon the supposition, that but one dose of the poison had been given. He replied in the affirmative, and added, that after a portion of arsenic has been absorbed, either an individual rejects all the remainder, or, what is more commonly the case, a portion of the fine powder adheres for several days to the walls of the stomach, in spite of the vomiting; thus, maintaining the irritation in that organ, and continuing to be absorbed.

In the *fourth* case, M. Orfila detected arsenic after preceding chemists had failed; and he makes the following observations on an occasional cause of failure.

"I will here make an observation, to which I attach great importance. I strongly protest against the practice so frequently followed, and so long advised by writers upon medical jurisprudence, viz., the *multiplying unimportant experiments*, for the discovery of poison in a suspected body. The fluids are treated by a variety of tests, when it would be possible to detect the poison by confining ourselves to one or two operations. By this proceeding a large quantity of the fluids are consumed, while the precipitates thus obtained have little or no value, and a sufficiency often does not remain to make the experiments, whose result alone can be conclusive. The impurity of many of these tests may also lead to fatal error.

Being consulted nearly daily by some of my brethren in the provinces, to whom the tribunals have confided these researches, I have become convinced, that much of the want of success arises from the causes I have mentioned."

The *fifth* case is that of the celebrated *Laffarge*, which has occupied public attention so much of late. This man was seized, on the 4th of January, with vomiting, colics, and painful constriction of the throat. These continued with more or less intensity for several days. He died on the 14th, and, upon examination, redness and ecchymosis of the stomach, and an ulcer of the duodenum, were discovered. In a first series of experiments, the tube broke while the reduction of the precipitate was proceeding. In a second series, a portion of the stomach was submitted to Marsh's apparatus, as also the matters vomited, and from neither was arsenic produced. In a third series, the body was exhumed, several chemists examined it by the processes recommended by Orfila, and with the same result. A fourth series, were conducted personally by Orfila, and he obtained arsenic from the stomach and its contents, as also from portions of the thoracic and abdominal viscera.

After the conviction of Madame Laffarge, a paper war was waged between MM. Raspail and Orfila. The former accuses Orfila of determining at all hazards to discover arsenic, and of having taken it with him in the nitrate of potass he employed in his experiments. In answer to other more reasonable objections of M. R. and of Madame Laffarge's counsel, Orfila observes—

1. That arsenic was not obtained from the muscular substance, because so small a quantity was experimented upon. 2. That the spots produced were truly arsenical, and in a greater number than was required to produce conviction. 3. That the preparation of iron which Laffarge had taken prior to death, could not be supposed to have contained arsenic, and if it did, no portion of it was found in any of the organs operated upon. 4. That the arsenic could not have entered his system while Laffarge was engaged in his foundry, as he had not been near it for fifty days prior to death; and if a small quantity in the form of vapour might have then obtained admission, a few days would have sufficed to have liberated it. 5. It is not true that arsenic is generated in any of the soft tissues during putrefaction.

It will be seen that this process of M. Orfila is one of considerable difficulty, as his directions were followed on two separate occasions by several chemists, and yet he alone was able subsequently to detect the presence of arsenic; and upon the strength of his evidence alone was Madame Laffarge condemned.

### III. Ligature of the Common Carotid Artery, for an Erectile Tumor of the Orbit, By M. *Jobert.*

M. æt. 60, came to Paris to consult the leading medical men, respecting a pulsating tumor, which, from being scarcely visible, increased in a few months to the size of an egg, and mounted up from the orbit on to the frontal bone. Insupportable pain attended every movement of the eye, while vision on that side was destroyed. All remedies having proved ineffectual, it was determined to tie the right common carotid artery. Immediately after the application of the ligature, all pain and pulsation ceased. The wound healed by the first intention, but the ligature, detained by the cicatrix, did not come away for a month. The eye which had projected now returned within the orbit, and its various movements were performed without pain or limit. Little or no pulsation was perceptible in the arteries of the face on the right side, but on the left side they were anormally developed, while the healthy eye was unusually brilliant.

In this case, and in another which occurred to the author, no *cerebral* symptoms supervened upon the ligature of the common carotid: but, finding a great discrepancy of opinion in the works of various writers upon this point, he instituted several experiments upon animals. The result of these was, that the tying the carotids was followed not by the production of cerebral mischief, but by the indications of a true pulmonary apoplexy; and, moreover, that this operation might be performed with impunity upon the dog, sheep, rabbit, and calf, but was fatal in the *horse.* In this animal, the vertebral arteries, large on entering their osseous canal, become almost filiform before penetrating the cavity of the cranium; and thus, after the ligature of the carotid, the blood not passing to the neck, head, and brain sufficiently freely, large apoplectic congestions of the lungs are formed. Bleeding, prior to and subsequent to the operation, was found to diminish the gravity of its effect, and M. Jobert suggests, that, in strong men, depletion should be resorted to, to prevent any pulmonic stasis.

### IV. Aneurism at the Origin of the Left Carotid treated by Ligature on the Distal Side. By M. *Colson.*

F. Jaunet, æt. 63, applied for a pulsating tumor projecting below the left clavicle. A ligature was passed around the left carotid on the distal side of the aneurism with some difficulty, as the tumor thrust the axis of the vessel between the transverse processes of the vertebræ and the posterior border of the sterno-cleido mastoid. The same evening, some feverishness being present, 12 ozs. of blood were abstracted, and this was repeated the next day, making the fourth venesection, as she had been bled twice prior to the operation. She went on well for several days, with the exception of the wound, which yielded pus of a serous quality. On the 20th day, a slight transudation of blood proceeding from the wound, and some fever being present, she was bled for the fifth time, and the blood taken was buffed. On the 28th day the ligature came away easily. On the 48th day another slight hæmorrhage and a sixth venesection. After this she went on well, and was about to be discharged cured, when a rapidly destructive ophthalmia attacked the left eye and vision was lost; 75 days after the operation the wound closed. About this time the woman fell down, and an increase of size and violent pulsations were perceived in the tumor; inflammation and threatening of abscess followed, but were soon relieved. Six months after the operation she was considered as cured, being relieved of all urgent symptoms, although a large swelling, and considerable pulsation remained.

This M. Colson states to be the 13th case on record in which the operation of Brasdor (the placing the ligature on the distal side of the aneurism) has been performed, and only the second instance of cure, the first being a case treated by Bushe.* But surely it is premature to pronounce this case, operated upon in January as cured in July.

### V. LIGATURE OF THE COMMON ILIAC, FOR AN ANEURISM OF THE EXTERNAL ILIAC. By M. *Deguise.*

Retouram, a muscular subject, applied on account of a pulsating tumor situated below the horizontal ramus of the pubis. The operation being decided upon, an incision was carried from below the anterior superior spinous process of the ilium to the outer border of the abdominal ring: care being taken to avoid the peritoneum, the sac of the aneurism was opened, an assistant at the same time, by compression of the aorta, preventing an undue flow of blood; a ligature was passed around the external iliac about an inch above the tumor, close to the common iliac trunk. Although the constriction of the ligature was made carefully, it divided the artery, and blood flowed abundantly, and another was therefore passed around the common iliac. The opening of the sac, and the division of the external iliac, rendered a ligature below indispensible; but, as the tumor occupied the whole of the external iliac, it became necessary to tie the femoral below the crural arch. In accomplishing this, the femoral vein was wounded, and it, as well as the artery, tied. The ligature of the iliac fell off on the 15th day, and that of the femoral on the 21st day. In spite of all the untoward circumstances connected with these operations, the patient proceeded on uninterruptedly to recovery.

### VI. DISARTICULATION OF THE HUMERUS, REMOVAL OF THE SCAPULA, &c. By *Gaetani Bey.*

Bedui-Hassanem, while employed in a foundry at Cairo, was struck by portions of a cannon which burst. By the blow, a portion of the scrotum was torn away and the cord divided, but the principal violence was offered to the arm and shoulder, which parts were shattered and lacerated

* See Lancet, Vol. XIV. p. 149.

in the most horrible manner. The arm was removed, but the scapula was so much injured that it was resolved to remove it also with its adherent muscles. The clavicle was promptly separated from the acromion, and a small portion of skin which still covered the inferior angle of the scapula carefully dissected off. The acromial end of the clavicle was afterwards sawed off, and the few remaining portions of the integuments brought together as nearly as possible by sutures. Some vicissitudes attended the case in its progress, but in two months the lad was discharged cured.

### VII. On Menstruation. By *A. Brierre de Boismont.*

This essay, designed to shew the influence menstruation exerts upon diseases, and how it, in its turn, is influenced by them, obtained the prize of the Academy of Medicine in 1840. The author divides the subject into two sections, a physiological and a pathological.

### A. *Physiological Section.*

The author believing that exact knowledge respecting menstruation can only be acquired by statistical enquiries, has conducted these among 1200 women of different classes of society.

A. *First Appearance of the Menses.*—Of 276 women living in the country, the mean age was 14 years 10 months; of 205 living in towns, 14 years 9 months; and of 359 either born in Paris, or who had lived there at least a year prior to menstruating, the mean age was as follows; viz. in 171 poor women, 14 years 10 months, which agrees with MM. Marc, Despine and Bouchacourt's statements; in 135 of middling classes, 14 years 5 months; in 32 girls in M. Bouvier's orthopædic establishment, 14 years 8 months; and in 53 of the highest ranks, 13 years 7 months. Chomel, Andral, and Recamier likewise fix the age for commencing menstruation among the upper classes at between 12 and 14. The average age obtained by the union of 359 of all classes in the capital is 14 years 6 months. Marc d'Espine places the mean figure in Paris at 14·965. It is at Manchester 15·191, and at Marseilles and Toulon 14·015. Many circumstances besides mere climate contribute to the difference, such as condition in society, nature of occupation, customs, manners and habits, education, &c. Dr. de Boismont has found that a very carefully superintended religious and moral education has the effect of retarding the appearance of the menses among the upper classes. The influence of individual organization is difficult of appreciation: an approximation in reference to *temperament,* among the lower classes may be obtained, and these may be arranged in the following order, beginning with that in which menstruation appears earliest:—sanguine, lymphatico-sanguine, lymphatico-nervous, and lymphatic. A robust state of the constitution favors the appearance of the menses, a delicate one retards it. Those who have fair hair are more backward in commencing than those who have dark, as are women of tall stature compared to the shorter.

Of 645 women, 357 commenced menstruating without, and 288 with more or less serious premonitory suffering.

B. *Establishment of the Period.*—Among most women menstruation is usually established by the sole efforts of nature, and mischief frequently results from injudicious interference with remedies when it is a little delayed. Of 70 women the average period between the preliminary symptoms and full establishment of the flux was 1 year and 4 months. After the first menstruation, the periods or quantity may be at first very irregular: thus, of 122 women the mean period required for the definitive establishment of menstruation was 1 year and 6 months. Of 654 women 65 have never menstruated regularly, but of these only a third part have suffered inconvenience from this, and those who did suffer were usually of a scrofulous, lymphatic, or ricketty habit of body. The mere irregularity, unless accompanied by symptoms, is not a cause of inquietude, although women subjected to it should be more than ordinarily careful in all hygienic precautions. As to the period of return, 28 days are usually supposed to elapse, and in a great number examined 30 days were found to intervene. The exact day is sometimes observed, and the most ordinary deviation is for this to be anticipated by some days at the next epoch. The discharge usually first appears during the day, especially among women engaged in active occupations, but among the delicate the night is generally the time—while with many women the time of day is very variable and uncertain.

C. *Duration.*—Of 562 women, the period of duration was in the following singular order:—in 172, 8 days; in 119, 3 days; in 78, 4 days; in 62, 2 days; in 46, 5 days; in 35, 1 day; in 21, 6 days; in 12, 7 days; in 17, above 9 days. The menses lasted long especially among the inhabitants of towns, delicate nervous women, and such as led indolent, voluptuous lives. Several women are equally unwell the whole time, while others are more so at the commencement, the middle, or the end of the period.

D. *Influence of Great Towns.*—It is very common for young girls who leave the country to live at Paris, to find an immediate diminution or suppression of the menses, while in a small number they are encreased. Often no unpleasant symptom attends this change, but at other times there is colic, griping, &c. The author is certain that a residence in the wards of a hospital has a tendency to produce a suppression. In young women, too, who came to the boarding schools and religious houses, often several months elapse before the re-establishment of the menses.

E. *Influence of Marriage.*—Of 25 women whose periods were irregular prior to marriage, in 14 they became regular. The author has seen several cases in which the menses appeared in the 2nd, 3rd and 4th months of pregnancy, *and three in which they returned regularly during the whole period (?)* Although usually the menses are suppressed during *lactation*, yet, in 15 cases they appeared during some portion of this period, and in 12, continued throughout. In most of these the children did not seem to suffer; but in one woman who menstruated every three weeks, and whose milk was of a bad quality, the child perished; and in another case in which the mother had menstruated during eight pregnancies, the milk was serous, and the children, who were very feeble, all died between the 4th and 5th year. Of 164 woman who were examined as to the effect of *delivery*, in 82 who spoke positively, the usual period of the return of the menses was from six weeks to two months; and when the return is delayed for several months (unless suckling) uterine affection frequently exists and should always be suspected.

F. *The Menstrual Fluid.*—M. Bouchardat undertook a new analysis of the menstrual fluid, obtained from one of our author's patients, who consented to allow a speculum to remain in her vagina for 10 hours, to obtain an ounce—a disagreeable and painful experiment, says M. De Boismont, and a somewhat revolting one, we may add. Without this precaution, the fluid becomes mixed with vaginal mucus and urine, as the presence of ammoniaco-magnesian phosphate proves. The following is the analysis :—

| | |
|---|---|
| " Water | 90.08 |
| Fixed Matters | 6.92 |

The fixed matters were thus composed :—

| | |
|---|---|
| Fibrine, Albumen, and colouring matter | 75.27 |
| Extractive matter | 0.42 |
| Fatty matter | 2.21 |
| Salts | 5.31 |
| Mucus | 16.79 |
| | 100.00 " |

The great proportion of water he considers as due to the delicate frame of the patient, and to her subsisting chiefly upon a vegetable diet.

Another specimen of menstrual fluid examined by M. Donné, gave the following microscopic characters ;—

" 1. Abundance of the ordinary globules of the blood.
2. Vaginal mucus formed of epidermic squamæ from the mucous membrane of the vagina.
3. Mucous globules furnished by the neck of the uterus."

From these examinations, it results that the menstrual flux does not differ from arterial blood. As to the acid or alkaline nature of the fluid, observed by authors, this depends upon the presence of mucus from the vagina and neck of the uterus. This mucus, as M. Nauche has proved, is acid in a healthy woman, and after delivery, but becomes alkaline when it is glairous or the product of inflammation : if only a limited portion of the passages be affected, the secretion will be acid and alkaline in different parts.

G. *Cessation of the Menses.*—In 181 women very different epochs were observed. Very rare in early life, cessation becomes frequent towards 40, and from thence to 50, very common, while after that age the number gradually diminishes. As to the entire duration of the menstrual life, it varies from five to 48 years, but the mean period is from 27 to 28 years.

Of 154 women, all symptoms or ill effects of the cessation at once disappeared in 40; and in 80 they had a mean duration of two years; in 12, the period was not precisely marked, and in 22 the symptoms still continued. *Metrorrhagia* followed the cessation in 57 cases. In moderation this is not to be feared, as it is often attended with but very slight debility. It is only dangerous when it indicates organic disease. It is much more frequent than supposed, and the author has repeatedly known it attributed to disease of the womb, and yet, after its continuation for several years, the woman has been restored to perfect health.

H. *Influence of Leucorrhœa.*—Observations respecting this were made upon 273 women. Of these 63 had leucorrhœa prior to the first menstruation, and several found it cease just prior to or during the first epoch. Two-thirds of these were born in Paris, and of fair complexion, and most of those who resided in the country were of scrofulous habit, but some others were healthy. In 248 women, leucorrhœa has appeared a varying number of years after the first menstruation, and in some, in whom it had existed prior to menstruation, it became increased. In 155, the leucorrhœa appeared just before the menses, was masked by them, and reappeared when these had ceased. In 225 women out of 248 the leucorrhœa returned after menstruation ceased. When leucorrhœa exists prior to menstruation, it retards the arrival of this more than any other cause, and frequently in these subjects the menses do not appear until 18 or 19. Chlorosis is then often present, and the constitution delicate. Marriage more frequently increases than diminishes leucorrhœa, and in many cases produces no effect. It is very common after delivery, and especially after abortion. It is common in young girls, who coming to live in towns are liable to irregular menstruation; in amenorrhœa it is very frequent, and would seem in some measure to supply the defective secretion. In its duration, symptoms, and extent it much resembles the menses, generally preceding and following them, but not as Baglivi thought, ceasing in the intervals: but the irregularities are too great to allow us to consider it as replacing the menstrual secretion.

### B. *Pathology.*

A. *Amenorrhœa.*—This may be—1. *Primary,* as when at the proper period the secretion does not appear: when, however, no symptoms present themselves no means need be adopted; but when the flow is impeded by a congested condition of the uterus, depletion and baths will be found very useful. 2. *Local,* when an occlusion of the uterus or vagina exists. 3. *Secondary,* or *amenorrhœa from suppression.*

Of 190 cases, the causes of suppression were physical in 68, moral in 92, and unknown in 30. Of all *physical* causes, the operation of *cold* is the most frequent, although habit may overcome this, as is seen in the bathing women; while, in some idiosyncrasies, cold has provoked an excess of the discharge. A temporary amenorrhœa often succeeds inflammatory diseases, treated by depletion, and may also appear during the convalescence of typhus fever. Many medicines also produce it, and especially copaiba and cubebs. Circumstances often modify liability to it in the same individual; thus, menstruation was suppressed in a lady by the application of a few leeches to the epigastrium, while the loss of an enormous quantity of blood from 160 leeches, by the same person, did not, on another occasion, produce the same effect.

In the treatment recommended, the author relies chiefly upon local and general depletion and warm baths. He thinks, however, other means may be necessary; but, he would seem to be little aware of what these are, since he quotes as a novelty a case in which the *pil. aloes c. myrrhâ* was given with advantage.

B. *Dysmenorrhœa.*—This is chiefly found in towns, and Lisfranc states, that it is often hereditary. Our author conveys no additional information respecting this disease, but he has some interesting observations upon some of the ailments afflicting the inhabitants of *convents*. The life of the cloister only commences after menstruation is established, but it is very rare not to find after some years a notable diminution in the quantity of the secretion. The period is usually very exactly observed, but with most nuns a mere shew for 24 hours occurs, which, however, slight as it is, seems necessary for their health. Their digestive organs are never in a proper state. There are no acute or gastralgic symptoms present, but rather an inactivity of stomach, accompanied by a sensation of general debility and sinking in the epigastric region. These women, full of the best intentions, are continually struggling against physical debility.

Tonics and stomachics are useless, and often hurtful. A slight papillary redness of tongue gives rise to the belief in the existence of a sub-acute gastritis, or a hyperæmic irritation of the mucous membrane of the stomach, and laxatives, alkalis, and rhubarb, are found very useful. This state of the digestive organs alternates commonly with leucorrhœa, and experiences a sensible remission during, and a few days after, the menstrual period. Vicarious hæmorrhage, and especially hæmoptysis, is often present, from which the general health suffers little, and tubercle does not follow. The nuns are also very liable to diarrhœa, otorrhœa, and obstinate cutaneous affections. Headaches are frequent, and are relieved by purgatives and snuff-taking. These women, although in a bad state of health, frequently live long, and are not liable to acute disease or cancer. Phthisis, however, especially in those convents in which the discipline is severe, carries off a great number.

C. *Chlorosis.*—There are many reasons to believe that chlorosis is a general affection, produced by changes in the quantity and quality of the blood, and analysis proves this fluid to be defective in cruor and iron. Other facts point to the nervous system as being primarily affected; thus, the division of the pneumo-gastric nerve defibrinates the blood, syncope lessens its power of coagulation, and almost any moral impression modifies the sanguification.

It would seem that occasionally chlorosis is a local affection which re-acts upon the general economy; thus, there are young persons in whom menstruation, which has been in vain solicited by tonics, iron, &c. appears immediately after marriage or pregnancy. Other girls presenting all the signs of chlorosis are at once cured by the mere local stimulus of sexual intercourse; while, in other cases, the affection seems to follow unavailing efforts at menstruation. Although chlorosis often accompanies amenorrhœa, yet the two are not necessarily co-existent; for, besides the fact that chlorosis occasionally occurs in man, so also it may be found in very various states of the menstrual function. It cannot always be said to be asthenic, for it sometimes occurs in females possessing all the attributes of power.

D. *Vicarious Menstruation.*—This is chiefly observed to proceed from the mucous membranes and the skin. It is seen as often in the plethoric as in the feeble woman. The stomach, lungs, and hæmorrhoidal vessels are those which are especially liable to become affected, although any feeble organ, or one to which irritation has been applied, may suffer.

In early life the upper, and later in life the lower parts of the body are those which are usually attacked. The discharge of blood generally appears a day or two prior to the menstrual period, and continues as long or longer than it. Sometimes it slightly co-exists with the menses, when the latter are then but a sanguinolent serum. The suppression of the vicarious discharge must be conducted very cautiously; and, when in a young person the health does not suffer, we must seek to do this only by means calculated to restore the natural menstruation. A mere expectant treatment would, however, not be right if a large flow of blood proceeds from the lungs or stomach. In our endeavours to establish menstruation, bleeding in the foot will often be useful, or, if general plethora be present, from the arm. Leeches to the vulva are very useful. M. Jamin recommends that depletion should only be employed some days after the cessation of the periodical hæmorrhage, in order to avoid any risk of sudden suppression.

E. *Menorrhagia.*—Women living in towns are most liable to this affection. Its danger must not be estimated so much by the quantity of blood lost, as by the comparison of this with the power possessed by the woman.

It is often seen in the newly-married, and especially when intercourse occurs near the menstrual period. It exposes women to abortion, and may lead to organic disease of the uterus.

The various uterine affections occur especially at certain *periods of life.* Thus, at (1,) the epoch of puberty, we find amenorrhœa and chlorosis, which may precede the flow, and these may be followed by dysmenorrhœa or vicarious hæmorrhage, but menorrhagia is then uncommon. In the second, or uterine period, suppression is frequent, but dysmenorrhœa is rarer, especially among the married, as are also chlorosis and vicarious hæmorrhage; but menorrhagia and metrorrhagia are commoner. In the third, or critical period, menorrhagia and metrorrhagia are frequent, vicarious hæmorrhage occasional, and chlorosis very rare.

F. *Influence of Menstruation upon Diseases.*—This is sometimes beneficial, but the menses appearing at the commencement of the disease have occasionally aggravated it; while immediate relief has often followed their appearance as a disease arrived at its height. In chronic disease, especially of the brain, the appearance of menstruation has likewise seemed frequently beneficial, although it is difficult always to say, whether it is the cause or consequence of the removal of the complaint. The beneficial influence of the *first* menstruation in removing many of the ailments of young girls, and procuring a robust health, is well known. Many affections, such as glandular disease, swellings of the joints, hæmorrhages, incontinence of urine, obstinate cutaneous disease, and especially various nervous diseases, &c. then disappear. On the other hand, it is sometimes associated with the development of grave disease, especially hereditary, as phthisis, syphilis, &c. In this case it is but the occasional cause of the manifestation of a latent predisposition.

The influence of the *cessation* of menstruation upon disease is indeed important, and may manifest itself in various modes.

1. Local Maladies.—Hæmorrhages and the various forms of leucorrhœa are very common, either as resulting from the presence of organic disease, or from a mere congested state of the uterus. Discharges have existed in some cases from 3 to 9 years, and yet have been unattended by organic disease. Cancer and other formidable affections are however but too common, and the author attributes the production of these, in many cases, to too early marriages, and to the too frequent, and too nearly the menstrual epoch, employment of sexual intercourse.

2. General Diseases.—Plethora manifests in various manners upon the different organs, and thus the neuroses, cutaneous diseases, hæmorrhoids, obstinate constipation, dysuria, &c. are produced.

3. Influence on existing Diseases.—The influence of the cessation in developing any existing germ of disease is well known, and there is scarcely a malady which may not become exasperated at this period: yet, as Fothergill observed long ago, some women, who have long suffered from debilitating discharges, seem at this period to acquire a renewed vigour of constitution, and from having been very thin become fat.

4. Proportionate Mortality from Uterine Disease at this period.—Of 721 women there were 373 who had had children, and 81 who had miscarried; of these about a third part had some functional or structural affection of the womb. Many of the women were unmarried—a class of persons peculiarly obnoxious to puerperal disease. It is certain then that after delivery the womb is liable to various affections, and the critical period of life seems also to arouse these. Of 60 women suffering from uterine lesions, in 35 these came on during the uterine or active period of life; a great majority of these lesions were of an inflammatory nature, and only four cancerous. Women are not aware of the liability of the uterus to become affected after delivery; for nearly six weeks it remains encreased in size and engorged, so that, errors in regimen, too early exercise, or too early sexual intercourse, may develop much mischief. Of 25 cases occurring at or after the critical period, 14 were cancerous, and 9 engorgements with discharges. In 6,825 women Madame Boivin observed 409 cancerous affections of the uterus, 203 of the ovary, and 56 of the breast, in all 707. Dionis declares that in 15 out of 20 women the affection only appears at this age. The greater number of women in 57 cases of polypi observed by Dupuytren were aged from 40 to 50.

It is very important to remember that 9 out of 25 of the uterine lesions were mere engorgements; and even affections feared to be mortal have been sometimes cured after a protracted struggle. The author says that women among the higher ranks, and especially the English at Paris, indiscriminately regard all ulcers as fatal, and thus allow honorable but dangerous scruples to prevent their seeking advice.

G. *Influence of Diseases upon Menstruation.*—1. *Acute.* There is scarcely an acute affection that does not derange menstruation, rendering it irregular and usually suppressing it. This is especially the case with inflammatory affections of the abdomen. As a general rule no special treatment must be adopted deriving the existence of the disease, although, occasionally, a speedy cure has resulted, by deriving the blood towards the genital organs. This, when desirable, is best brought about by depletion from the foot or arm or the use of an emetic ; baths, leeches, cupping-glasses, and a ligature, are desirable under various circumstances. 2. *Chronic.* Here too, the usual effect is suppression ; and so frequently does this occur in cases of phthisis, that the author thinks it may form one point of diagnosis between this disease and chronic bronchitis. Affections of the heart in their latter stages, of the alimentary canal, and of the liver, often cause suppression, and many lesions of the uterus only manifest themselves by changes in the function of menstruation. Affections of the nervous system, as hysteria and epilepsy, have a notorious effect upon the menstrual function. In insanity varieties in menstruation are very frequent, and, while in a few cases these precede, in the great majority they follow, the loss of reason. In 101 girls affected with distortion of the spine, M. Bouvier found 32 first commenced menstruating while under treatment, in 24 the menses continued regularly, and in 40 they were suppressed, but without serious symptom. When surgical operations are performed near the menstrual epoch, the menses are frequently anticipated, but when at a distance from this, they are often delayed. Occasionally after operations the wounds discharge blood periodically, and should not then be officiously meddled with.

### VIII. ON HANGING. By M. *Orfila.*

M. Orfila, after detailing the particulars of a very interesting case of a man, who was first stifled by his wife and son, and then suspended by a cord, thus reviews the opinions generally entertained as to the signs which distinguish, whether suspension has occurred prior to or subsequent to death.

1. *It is said that in persons hung during life the face will be found livid and swollen ; the lips in the same state, and twisted : the eyelids swollen, half-closed, and bluish ; the eyes round, and prominent.*

These characters are often wanting in persons who have really hung themselves, while they have been found to exist in other cases where suspension has not occurred until after death.

2. *The tongue is swollen and livid, impressed by the teeth and frequently protruded; a sanguinolent froth is found in the nostrils and gullet, and*

*around the mouth. The lungs, heart and brain are more or less gorged with blood.*

These signs are of no value, as they are frequently found wanting in persons hung during life, and may be produced by asphyxia from other causes.

3. *There will be ecchymosis from the impression of the cord.*

This is one of the most certain signs, as it has never been produced by hanging bodies immediately after death, although perhaps the experiments have not been yet sufficiently varied to enable us to pronounce from it alone. It is absent much oftener than supposed. Klein found it so in fifteen persons, Esquirol in twelve, Orfila in fifty, Fleischman in five, and Devergie in twenty-five persons, hung during life. So far from being ecchymosed, the subcutaneous cellular tissue, corresponding to the cord, is usually dry, pearly white, dense, and filamentous. A mark of the cord of a livid or black color, like burned parchment, is however always present, but without ecchymosis; and it may be produced by hanging after death, as Orfila shewed long ago in his experiments. There can be no doubt that this mark has often been described by authors as ecchymosis, and we thus see the rashness of the rule given by Belloc, Mahon, Foderé, and others, that, if the impression of the cord was red or violet, the suspension had occurred during life.

4. *Bloody excoriations of the skin, or ecchymoses among the cellular tissue of the neck, the subjacent muscles, or in the neighbourhood of the larynx.*

These are valuable signs, but seldom exist; and unless produced by blows during life or immediately after death, prove that the suspension occurred during life.

5. *Rupture of the muscles or ligaments of the os hyoides, or tearing of the larynx or first ring of the trachea.*

Morgagni, Valsalva, and Weiss mention these; but Orfila out of 50 cases has only once met with a fracture of the os hyoides. Moreover, by imparting varied twisting movements to bodies suspended after death, he produced a fracture of the thyroid cartilage but once.

6. *Congested state of the genital organs, and semen in the urethra.*

Our readers are aware that M. Devergie has announced this as a sure sign of suspension during life, and that M. Orfila has denied the correctness of the assertion.* Numerous experiments have convinced the latter observer that it is not uncommon to find semen in the urethra of many individuals who have died of various diseases, and especially if they have lain upon their backs; while, again, by suspending for a few hours the bodies of persons who had recently died, he produced congestion of the genital organs, and even erection of the penis; and in these, the existence of live zoosperms may be proved. In two cases recently observed by

---

* Medico-Chirurgical Review, Vol. xxxi., p. 551—Vol. xxxii. p. 203.

M. Ollivier d'Angers, in which the bodies were cut down shortly after suspension, a positive flaccidity instead of congestion of the genital organs prevailed.

7. *Rupture of the inner and middle coats of the Carotids.*

This is a sign of little value, for it occurs only rarely when hanging has occurred during life, and M. Malle produced it twice in experiments upon the dead body.

8. *Injuries of the Cervical Vertebræ.*

M. Orfila dwells much upon this sign, believing that it has not been sufficiently investigated, and that several errors prevail respecting it. He proposes six queries upon the subject.

A. Can we, by any violence employed on a suspended corpse, produce a luxation of the 1st or 2d cervical vertebræ?

In 20 experiments, made by employing violent flexion, extension and torsion, and by the application of the additional weight of other persons, no displacement whatever was produced, but a fracture occurred once.

B. By the same means can luxations be produced in other parts of the cervical region?

In these vertebræ the violence produced greater effects: one of the ligamenta subflava was torn, so as to expose the cord to view, and some of the intervertebral substances were more or less torn.

C. Can the first vertebra be dislocated from the second during an execution?

Although this is admitted as possible by most authors on medical jurisprudence, Orfila considers it very doubtful. The statement made by Louis of the executioner of Paris producing this luxation, by springing upon the body of the culprit, as he was turned off, and impressing a lateral movement upon it, is of little value, as that surgeon never assured himself of the fact by dissection. Orfila in his experiments has caused one, two, or even three men to mount upon the suspended body, and has submitted it to every possible movement, without ever producing this effect. So, too, when Petit explains the death of the child lifted up by the chin and occiput, by stating that the odontoid process passed from under its ligament, he does not state that he examined the body to prove whether this was the case. Surely if this accident were of such easy production as some believe, by reason of the slight development of the process in the child, and its slighter retention by the ligament, we ought to see it frequently, and not have to refer to this solitary case; for children are daily lifted up in the same manner. In the author's experiments, the bodies of children from one to four years of age were submitted to all sorts of movements without producing this effect, although in three out of four there was a separation caused between some of the inferior vertebræ of the neck.

D. Can a dislocation of the first from the second vertebra occur in suicide from hanging?

Orfila's experiments, which have embraced movements far more violent than those which a suspended person could employ, prove that this is impossible.

E. Can hanging during life (whether the effect of suicide or homicide)

give rise to injuries of the vertebræ of the cervical region, situated below the second?

M. Orfila, judging from his experiments, answers this query in the affirmative. It is true that the violence required in these to produce this result was considerable, but he can believe, in an aged, or delicate person, the exertion required for committing the act of suicide, may be sometimes sufficient.

F. Can we derive from the condition of the vertebral column signs sufficient to determine whether the suspension occurred during life or after death?

The state of the spinal column taken alone does not enable us to determine this. Although M. Devergie states that injuries to the vertebræ, spinal marrow, musles and ligaments, are, when accompanied by ecchymoses, signs of hanging having occurred during life, yet Dr. Christison has proved by experiment that all these lesions may be produced by blows inflicted upon the dead body. The various injuries alluded to may have been produced shortly after death, and prior to suspension. If there be fracture of the bodies or processes of the vertebræ, and especially if some of the lower vertebræ are luxated, and their ligaments torn, we must believe that the individual has been killed prior to suspension. Hanging, it is possible, may have occurred during life, but not from suicide.

9. Although the various cadaveric signs alluded to are frequently insufficient to determine the question embraced in this paper, a consideration of the various corroborative circumstances (*e. g.* the length of the cord, the manner of its application, the position of the body, &c.) will frequently enable us to affirm that the suspension has occurred during life.

IX. A very long essay upon *over-excitement of the nervous system,* is a most wordy affair, and we pass on to more interesting matter.

### X. History of the Discoveries relating to the Venous System from the Time of Morgagni. By *A. Raciborski.**

### A. *The Anatomy.*

The most interesting discoveries which have been made respecting the veins during the present century, are the existence of venous canals within the substance of the bones, and the venous circulation of the spine.

1. *Venous Canals of the Bones of the Cranium.*—It had been long known that an extensive venous communication existed between the external and internal parts of the cranium, but Dupuytren, in 1803, first demonstrated the existence of true canals. Chaussier and Fleury illustrated the same subject, but it was not until 1819 that Breschet published an exact description of them. They are distributed throughout the diploe, communicate freely with other veins, and are lined by a very fine membrane. A great analogy exists between the relations of these vessels to the diploe, and the disposition of the cells in the corpus cavernosum of the penis and

* This valuable paper obtained the prize.

clitoris, and if the substance of the diploe were fibrous rather than osseous, an exact idea of erectile tissue would be presented. This free circulation through the cranial bones explains the sympathies existing between the brain and its membranes, and the hairy scalp, as seen in the occasional effects of erysipelas, eruptive diseases, &c.—the inflammatory condition is propagated by continuity along these veins. Breschet found several of his epileptic patients, to whom the moxa had been applied on the scalp, die of meningitis; and he has often known children die with all the marks of inflammation of the brain, after the use of topical irritants to remove some affection of the scalp—and in all these the diploe was found red and gorged with blood. In the same way, many wounds, contusions, &c. affect the brain. The same occurs in other bones, for Cruveilhier found, in a patient who had sank from suppuration of the lungs supervening upon amputation, that the phlebitis had commenced in the spongy extremities of the bones of the leg. Necrosis of the bones of the cranium has originated in the same manner. This disposition of the veins explains the effects of leeches, cupping, &c. applied to the scalp in cerebral affections.

2. *Spinal Veins.*—The vertebræ are traversed by venous canals communicating with the great spinal veins. Breschet first demonstrated the beautiful distribution of these latter, which, from their size, seem almost analogous to the sinuses of the dura mater. They constitute a most important medium of anastomosis between the veins of the pelvis, spinal marrow, and pelvis, and indeed every important neighbouring organ in the economy. This junction of so many distinct parts explains many of the simultaneous changes in distant organs, hitherto explained upon the doctrine of sympathy; *e. g.* the occurrence of abscess of the liver after the injuries of the head, for the spinal veins may communicate on the one hand with the inflamed veins of the cranium, and on the other with the vena porta.

3. *Structure of Veins.*—Disputes have long occurred as to the direction of the fibres of the *middle coat* of veins; but Cruveilhier declares that they have none whatever, and that a vein only differs from an artery by the absence of this middle tunic: however this may be, all agree that when the veins enter the parenchyma of organs, they become deprived of all but their lining membrane. Thus, the cells of the corpus cavernosum, and the substance of the spleen, are only extensive venous plexuses, while a great portion of the structure of the uterus is likewise venous, and hence its liability to venous inflammation after parturition. Ribes states that the capillary extremities of the veins are continuous, not only with those of the arteries, but with the cells of the cellular tissue. And speaking of these capillaries, Cruveilhier observes, "they seem to constitute a vast reservoir, in which are transacted all the phenomena of nutrition, of normal and morbid secretions, and of inflammation; in which are deposited, with the products of absorption, all morbid matters that are engendered within, or obtain admission from without, into the economy. It is in their extremities that diseases, primarily local, become general. The arteries are but passive conduits of the blood propelled by the left side of the heart." The description of the *valves* by Fabricius left nothing to desire. The absence of this structure in the veins of the spine, cells of the spleen, &c. seems to permit the blood in these to flow in two directions, and enables these parts to become reservoirs of this fluid, capable of temporary removal from the influence of the general circulation. This disposition however shews the possibility of the reflux of pus from the veins of the diploe to the vena cava. The *vasa vasorum* of veins have now been amply demonstrated. As long as the existence of these was unknown, it was believed that veins inflamed with difficulty. The *sensibility* of veins, even upon irritation of the inner tunics, has been denied by Haller and Bichat, and maintained by Monro, Chaussier, and Longuet. Cruveilhier, denying the existence of fibres in veins, does not believe they possess *contractility,* at least farther than that derived from their outer coat, which is composed of erectile tissue similar to the dartos. M. Berard* first pointed out, that many of the veins are found to be attached to neighbouring fasciæ and aponeuroses, by which means they are, during the various movements of the body, maintained in an open state, and prevented collapsing. The vena cava superior is thus maintained by the fibrous prolongation of the pericardium, the vena cava inferior by a fibrous layer from the diaphragm, and the ramifications of this latter in the pelvis are alike supported by the fasciæ. Many other veins are similarly disposed, and if we add to these the sinuses of the brain, the veins of the spine, and the venous canals of bones, we shall observe that a very considerable portion of the venous system has the walls of its canals maintained continually in an uncollapsed state. This disposition of parts plays an important part in enabling the atmospheric pressure to become instrumental in aiding the circulation of the blood.

4. *Anastomoses of Veins.*—The anastomoses of the venous system are far more numerous than those of the arterial; and so especially is this the case with regard to the spinal veins, that if the venæ cavæ and the vena azygos were obliterated, still, through their medium, would the circulation be maintained. There are, in fact, two systems of the venous circulation: one, a superficial one, belonging to the external parts, and the various viscera; another, and deeper, in the interior of the bones, and the cavities of the cranium and spinal column. These two systems may be substituted the one for the other, compatibly with the continuance of life. In fact, as Cruveilhier observes, the entire venous system is a large vascular network, and the blood need not pass through any one invariable and determinate channel to arrive at the heart. There is no organ which may not be said to be in communication with every other organ—an important fact in the consideration of phlebitis.

5. *Anastomoses with the Portal System.*—These do not occur alone in the liver. Cruveilhier has frequently seen injections thrown into the vena cava penetrate the ramifications of the vena porta. Retzius injected the vena cava and vena porta with different colors, and found the colon and meso-colon to be injected with both—the injection coming from the vena porta being nearest to the mucous membrane. Breschet has injected the

---

* Archives Generales de Medicine, Tome 23.

inferior mesenteric by the vena cava. Schlemm has found communications at the anus between the inferior mesenteric and hypogastric veins. The free intercommunication between the two systems explains how easily disease may be propagated from one to the other, and this may be a reason why abscess of the liver occasionally follows inflammation of the general venous system, as, *e. g.* in uterine phlebitis.

6. *Anastomoses with the Arterial and Lymphatic System.*—The communications between the veins and *arteries* had been fully established prior to the epoch of Morgagni by the researches of Malphigi, Ruysch, Cooper and others; while Soemmering and Prochaska have proved that the mode of communication varies, not only in different classes, but in different organs. The communication of the veins with the *lymphatics* has also been long known, but the mode of anastomosis has given rise to much dispute. Many of the contemporaries of Morgagni maintained that direct communications could be demonstrated to exist between the venous and lymphatic trunks in the mammifera, as they have been since shewn in the abdominal viscera of fish, and the limbs of frogs, by Fohman, Müller, Panizza and others. But the opinion, which has prevailed amongst all anatomists of modern times (except Monro, Mascagni, and a few others) is, that the only anastomosis which occurs between these vessels takes place in the lymphatic glands.

7. *Venous System in the Embryo.*—M. Serres first shewed that the various stages of the development of the venous system in the embryo resembled the permanent condition of the same system in some of the lower animals. Thus, at an early period, the venæ cavæ are double, and separate, and, gradually approaching inwards from without, eventually form a common trunk. This condition is found a permanent one in the porcupine and elephant, birds, reptiles, and fishes. Veins, as observed by Meckel, are far less liable than arteries to anatomical anomalies, and when these do occur, they seem to be true arrests of development, corresponding to the normal condition of the lower animals, as the various examples of double venæ cavæ seem to prove.

### B. *The Physiology.*

1. *Venous Circulation.*—It has been too much the custom to attribute this to some one exclusive cause, and thus neglect a general view of the various circumstances which contribute to it. Dr. R. passes these in review.

A. *Influence of the left side of the heart, and of the arteries.*—The action of the heart was believed by Haller, Soemmering, and a host of able physiologists, to be the principal cause of the circulation in the veins; while others, seeing the circulation has continued even when the heart has been enfeebled or removed, have denied this. Among the inferior animals the communication between the two systems is so direct, that little force is required to propel the blood into the veins; but in the mammiferæ, and especially in man, owing to the resistance of the capillary system, and the numerous angles and curvatures, a far greater propelling force is required, and the experiments of Magendie and Poiseuille prove that the heart is a principal agent. The degree of impulse becomes modified by the retardation which takes place in passing through the capillaries, and by the increased capacity of the venous system : for, as Magendie observes, if the capacity of the arterial system be stated as 1 and that of the venous as 10, it is obvious that, as the blood enters this latter, its impulse must become diminished. Contrary to the opinion of Bichat, the arteries are also instrumental in impelling the blood, by reason of their elasticity, which also converts an intermitting into a continuous movement.

B. *Influence of the Capillary System.*—Some physiologists confer a very exaggerated and almost exclusive importance upon the capillaries. To explain partial changes of colours of the skin, the phenomena of inflammation, &c. Bichat, Beclard, Gerdy, Schultz, and others, believed that the influence of the heart terminated prior to the commencement of the capillaries, and every variety of hypothesis was invented to explain the mode in which these latter acted. The direct experiments of Poiseuille, Magendie, and Dubois prove that the capillaries do not contract, and hence they have no influence in the movement of the blood.

C. *Influence of the Veins themselves.*—If the veins have not coats thick enough to accelerate the circulation of the blood, there are many circumstances connected with them, which facilitate its progress, such as the directness of their course, the numerous anastomoses, the presence of valves, the conical disposition of the apparatus, whereby the blood is constantly passing from a larger to a less space, &c. Any active force the veins may be supposed to exert is due to their elasticity, and to this must be referred all that has been said respecting their contractility.

D. *Influence of contraction of Muscles.*—Any one who has performed venesection, must have remarked the influence of muscular action in accelerating the flow of blood ; and Haller ranked this as one of the propelling agents. The irregularity and inequality with which this power is exerted, prevent our considering it as indispensable to the maintenance of the circulation, while under some circumstances it might be the means of impeding it : the abdominal muscles and diaphragm, however, exert, by their regular action, a considerable influence.

E. *Influence of the right side of the Heart.*—This was denied by Harvey, but admitted by Haller, who yet found that it was not indispensible. Most physiologists who have admitted it, have endeavoured to prove an active dilatation of the organ ; whether such exists, or that it merely results from the cessation of the systole, it is certain that an aspiration or suction action exists, the effect of which is much increased by the walls of the principal veins being kept, as already observed, in a state of patency by their attachment to the fasciæ. Spallanzani, J. Müller, and Dællinger have proved by vivisections that the blood flowed far faster in the venous trunks during the diastole, but it is to M. Poiseuille that we are indebted for exact experiments in proof of this.

F. *Influence of Respiration.*—This has caused much discussion. Valsalva first observed it, while experimenting upon the jugular vein of a dog. Morgagni and Haller admitted it as one of the auxiliary causes. Barry, in 1825, revived attention to the subject, and he first proved the actual suction effect of the lungs, and attributed it to the isochronous dilatation of the mediastinum, and of the walls of the thorax. His theory of the almost exclusive effect of respiration has met with many objectors, who point to the fœtal state and to many of the lower animals, in which the circulation proceeds independently of respiration. This only proves that the agency of respiration is only auxiliary, and not so exclusive as Barry supposed: it cannot influence the capillaries, but only the larger venous trunks, which, as already observed, are maintained in an open condition by the various fasciæ. The experiments of Magendie and Poiseuille prove the limited extent and non-indispensible presence of this influence. The effects upon the abdominal circulation during respiration are due to the pressure of the muscles: the portal circulation is somewhat submitted to this suction action. The knowledge of the influence of atmospheric pressure is not without its applications; thus surgeons well know that in wounds of veins near the heart, the hæmorrhage is great in proportion to the difficulty of respiration; so too, when air has entered the veins during operations, it has done so usually after strong inspirations.

G. *Venous Pulsations.*—Although Poiseuille's experiments prove that during the flow of blood a slight jerking movement occurs in the vein, yet this is not seen through its coats. In an anormal condition venous pulsations have often been observed, (especially in the jugular vein) extending in some cases to the most distant extremities of the vessels. They must be distinguished from the movement imparted by contiguous arteries, or caused by the reflux of the blood in respiration. They have been accounted for in two ways—by the supposition that the impulse of the heart was anormally transmitted through the capillaries to the veins; or, more probably, by the reflux which occurs during the systole of the right ventricle. In almost all the subjects of this anomaly a dilatation of the right auriculo-ventricular orifice has been found, or some change in the tricuspid valve preventing its due application. Morgagni observed that in these cases there was always some lesion of the right side of the heart, usually dilatation of the ventricle. The experiments of Poiseuille and Magendie prove that it may be caused by a dilatation of the base of the venous trunks opening into the heart, and especially the external jugular, a dilatation which renders the adaptation of the valves at these parts impossible.

2. *Venous Absorption.*—The belief in venous absorption entertained by Haller, Meckel and Boerhaave, was not brought into question until about the middle of last century; when absorption was attributed exclusively to the lymphatic system by the Hunters, Cruikshank, Mascagni, &c. Morgagni partook of Haller's opinion, while Bichat believed in the exclusive functions of the absorbent vessels. The experiments of Magendie in our own day are well known. It is by imbibition that fluids pass from without to within the vessels, there being no specific absorbent mouths, as formerly believed. Imbibition is possessed in common by the veins and lymphatics, but, for absorption to take place, it is necessary that the fluid imbibed should be brought within the torrent of the circulation. The lymphatic vessels, being neither filled with fluid, nor traversed by a current, do not fulfil the necessary conditions. The doctrine of venous absorption has modified the theory of certain forms of dropsy, heretofore attributed to atony of tissue or general debility; these may now be frequently explained by an obliteration of the veins, while, as this obliteration frequently results from inflammatory action, antiphlogistic treatment is indicated in the early stage. We no longer believe in the existence of absorbent mouths having the power of electing substances useful to the economy, and rejecting noxious matter; but we endeavour to prevent the introduction of poisonous matters into the veins, or when this is impossible, to impede their transmission into the economy: hence the use of the cupping glass, and compression of the veins by a ligature. The experiments of Magendie and Bouillaud prove that the action of a poison may be accelerated or retarded, by relaxing or tightening ligatures attached to the veins corresponding to the part at which the poison was applied. Endermic medicine has derived its present expansion from our improved knowledge of the absorbent system.

### C. *The Pathological Anatomy.*

With the exception of a few remarks scattered through the writings of Morgagni and Valsalva very little knowledge concerning the pathological changes in the venous system existed, until the present time. Even Bichat had no idea of these affections which form so important a part of modern pathology.

1. *Redness of the inner Membrane.*—The inner membrane is frequently of a more or less deep red colour, but, when unaccompanied by vascular injection, this probably results from imbibition. The redness is deeper when the blood is in a putrid or very fluid condition. Inflammation may be present without the slightest redness, and, as a general rule, redness arising from this cause is less in extent and intensity than when resulting from imbibition; it is often dispersed in little patches or points.

2. *Secretion of Plastic Lymph and formation of Clots.*—The most simple form of alteration in secretion, is the exudation of a plastic lymph, analagous to that uniting wounds, and by which the canal sometimes becomes *obliterated.* At other times it takes the form of *false membrane,* which may more or less occupy the calibre of the vessel, or lie in layers without obstructing the circulation. These membranes are usually amorphous, but Ribes found them organized in one case. More frequently *clots* are found within the veins, uniting their walls more or less closely; they are sometimes several in number with portions of vein between them. The clots become deprived of their coloring matter and serosity, and fibrine, adhering to the walls of the vein, alone remains: this is sometimes transformed into an organic and vascular substance, commencing with the circumference. These changes take place only in veins which are secondarily obstructed by other clots, formed in the immediate vicinity of the inflamed vessel: in the latter, instead of an absorption of their various parts occurring, they become decomposed, and pus is generated. When the capillaries are affected, a greater volume or tension of the parts may exist, without any great obstruction of the circulation: but when a principal trunk is affected, the various ramifications become distended, a swelling is formed below the obstacle by the deposition of serum in the cellular tissue, and the capillaries may rupture and cause ecchymosis. When the sanguine concretions do not disappear with the other marks of phlebitis, they become an organized obstruction to the calibre of the vessels, and more or less infiltration occurs. Spontaneous *gangrene* arises from obstruction to the circulation; and, if it is the obstruction of the arterial system to which it is usually attributed, this is because the anastomoses of the venous system are so numerous that its complete obstruction is of rare occurrence.

Godin maintains that the source of the obstruction may be known from the nature of the gangrene; when this is moist, it is caused by impediments in the venous, and when dry by impediments in the arterial system; and, whenever in the course of a case of gangrene, humidity appears, the extension of the obstructing cause from the arteries to the veins is indicated.

3. *Pus in the Veins.*—The change of the clots into pus may sometimes be traced in all its phases. It commences at the centre; the pus is sometimes laudable, at others very dark, while it may be in so small a quantity as only to render the blood darker and more fluid. It is found in the ramifications of the parenchyma of organs, as well as in the trunks: large collections have sometimes formed between the valves, and given rise to all the appearance of a true abscess. The vein may burst in some parts, and numerous abscesses be formed along the course of its trunk. Sometimes the pus is infiltrated between the coats of the vein, or into the cellular exterior. Veins frequently contain pus after amputation.

4. *Coincident Affections of the Viscera.*—The coincidence of suppuration of the liver, with suppurating wounds of the cranium, having attracted attention, it was believed to be a pathological law. Valsalva and Morgagni first limited the number of these cases, and proved that the liver was not the only organ liable to be so affected. The depositions of pus are more frequently found in the lungs and liver, although they are seen occasionally in various other organs. The walls of these abscesses are sometimes naked, at others lined with soft false membranes. The tissue of the organ in which they are situated often seems little injured. Many explanations of these occurrences have been offered. The most ancient and general, is that given first by Morgagni, viz. the transportation of the pus by the veins from the suppurating wound to the affected organ. But we now know that when pus is found in veins, it is formed primarily within them, and has not its source from without. John Hunter first described the state of phlebitis in veins after amputation; and since that period, a crowd of celebrated men have published upon inflammation of the veins. In 1819, Breschet published, in his valuable translation of Hodgson, many examples of phlebitis; but he does not notice the frequent coincidence of visceral abscess and solutions of continuity. Dance was the first person in France who expressed himself as satisfied that the pus proceeding from the phlebitis, and mingling with the blood, was the cause of the production of the other special inflammations. His opinion has been adopted by Blandin and Cruveilhier, and is very general at the present day. Objections to this theory have been frequently offered, especially its application only to some of the cases: thus, Velpeau met with 13 cases of these visceral abscesses, in which no phlebitis was discovered. Tessier has met with similar cases, and observes, that even if phlebitis were present the resulting pus, blocked up by clots, could not become mingled with the blood. Tessier explains all the lesions which succeed to wounds and delivery by the existence of a purulent diathesis, but to this there are also objections, for persons in whom these lesions occur, prior to their appearance, often manifest no signs of bad health. Dr. Raciborski suggests that all the phenomena in these cases, like the analogous ones of variola, glanders, and the various eruptive fevers, bear a strong analogy to the chemical process of fermentation. A small portion of the febrile poison, introduced into the blood, generates an immense additional quantity of poisonous matter. He considers that the poisonous matter in the present cases is pus in a state of decomposition; and, adds, that although most veins are plugged up, as Tessier observed, this is not the case with the veins of the bones and those of the uterus. In these, complete obliteration rarely exists, and it is to be remembered, that it is especially after amputation, and after delivery that these affections occur. Often, too much importance is attached to anatomical lesions, and, in this way, a change really due to the primary change in the blood itself may be improperly attributed to phlebitis. Thus, wounds from dissection have been attributed to phlebitis, and yet the very cauterization, so useful in relieving them, would but increase it. Phlebitis does not exist, but, by the cautery, we succeed in chemically destroying the matter which is in a state of decomposition. If it does occur in these cases, it is only consequent upon the altered condition of the blood. So, in glanders, in which purulent deposits, so analogous to these we are considering, are produced, phlebitis has not been discovered to exist.

5. *Air in the Veins*—Morgagni explained the occurrence of death from the injection of air into the veins, by the consequent distention of the heart preventing its contraction—analagous to retention from distended urinary bladder; and it is certain that in most animals, which have perished from this cause, the right side of the heart has been found filled with frothy blood, and distended with air. Magendie, Dupuytren, Delpech, &c. have shewn that the accidental introduction of air into the veins must occasionally be considered as a cause of death during operations. As it only follows operations about the neck, the suction power of the lungs, acting through the veins which are maintained in an open condition by the fasciæ, has been assigned as the cause of the entrance of the air. The experiments of M. Amussat, in 1838, threw much light upon this subject. Of 26 animals, in whom a vein of the neck was opened, all died shortly with the exception of two, and the same auscultatory sounds and post-mortem appearances were observed, as are found in men who have thus perished during operations. The abstraction of a small quantity of blood renders the death only more speedy. These experiments proved that an open state of the veins is indispensable, and when this does not exist naturally, it may frequently be induced by some pathological cause, as the adhesion of tumors, &c. The same effects resulted from operating on other veins besides those of the neck, providing they were maintained open artificially, and this may explain some cases of sudden death in parturition.

It also results from these experiments, that death is sudden in proportion as the introduction of air is forcible; when this is not the case, death is not an indispensable circumstance, and many cases are on record of the alarming symptoms produced by this accident gradually disappearing.

### D. *The Pathology.*

1. *Phlebitis.*—Phlebitis may be produced by any irritation acting directly or indirectly upon the coats of the veins, as, solutions of continuity, ligature, phlegmon of various organs, &c. Ribes gives a case of phlebitis in the arm from chilblains on the hand. Cruveilhier and Fozeau, two cases of hepatic phlebitis from inflammation of the intestinal tube and biliary ducts. Andral and Bouillaud cite similar facts. Metastasis has been accounted a cause also, of which a remarkable example was offered in a person, who, afflicted with a chronic pemphigus, dried it up with some quack ointment, and, in forty hours, was seized with typhoid symptoms and died. Upon examination, the vena cava was found injected, and the structure of its inner membrane materially altered in texture. There are several varieties of phlebitis.

A. *Traumatic Phlebitis.*—(1). *From Venesection.*—This serious accident is especially liable to occur in some constitutions. Hunter looked upon the suppurating condition of the edges of the puncture, which is more likely to occur when these are not well closed, as very favourable to its production. The inflammation usually comes on in a few hours, follows the course of the blood, and sometimes extends to the heart itself. In two or there days, a little pus may be forced from the vein. The constitutional irritation is great. By reason of the anastomosis the circulation is not impeded, but considerable temporary contraction of the arm may occur, from the spreading of the inflammation to the fasciæ and cellular tissue. When a large portion of the veins of the part are affected, or the suppuration is abundant, the case often proves mortal. Sometimes, all the symptoms of the most terrible prostration occur from very inadequate local mischief, and then probably depend upon a change to which the whole mass of the blood has been subjected. Such symptoms are occasionally not manifested until after cicatrization has occurred.

(2). *After Amputation.*—Hunter first described this, and attributed to it much of the general febrile irritation occurring after amputation. Blandin has remarked, that it especially affects the small veins. How are these accidents produced? By irritating the veins by the application of the knife, ligature, dirty instruments, &c.? How happens it, then, that irritating substances may be injected into the veins without producing scarcely any ill-effects upon the lining membrane? The various changes probably arise from a primary affection of the blood, produced by the introduction into it of matters in a state of decomposition; whether this arises from the employment of foul instruments or dressings, or from their presence in a tainted atmosphere. The ill-effect is confined to the course of the blood, and does not manifest itself in a double direction. The small puncture of venesection, or the ligature of veins after operations, will never account for the general symptoms which result. The blood, once changed by contact with matters in a state of decomposition, the pus produced by the consecutive phlebitis, will doubtless render the symptoms yet more severe.

B. *Umbilical Phlebitis.*—This has been noticed by Meckel, Sasse, and Osiander; and M. Duplay has lately published several cases. Usually, from the second to the 4th day, but sometimes as late as the eighth day, the child is seized with violent colic, distension of the belly, &c.; the skin often becomes icteric, &c. the mucous membrane of the mouth is reddened: erysipelas is frequent, and usually commences at some part distant from the umbilicus. Death takes place from the 4th to the 14th day after birth. Besides marks of inflammation in the umbilical veins, the same have been found in other organs, as the pleura, the peritoneum, the lungs, the joints, &c. This suppurative form of the inflammation is very rare, while the adhesive form is almost as common as the ligature of the cord; and to the consecutive excitement of the liver by this inflammation, the author is disposed to attribute many cases of jaundice in new-born children. Andral and Ribes cite cases of jaundice in the adult, which were produced by a phlebitis extending from the upper portion of the digestive canal to the liver, through the branches of the vena porta.

C. *Puerperal Phlebitis.*—Phlebitis occurring after delivery is the most important form of the affection, and may be divided into several varieties.

(1). *Adhesive Uterine Phlebitis.*—According to the author, there can be no delivery without more or less adhesive phlebitis. (!) Usually it is confined to the portion of the uterus, where the placenta was situated. He agrees with Cruveilhier in considering a woman recently delivered in an analogous condition to one who has undergone a surgical operation, or received a wound, and that the consequent re-action in both cases, often in the one attributed to milk fever, is produced by the consequent phlebitis. The period of the appearance of the fever, and the fætor of the discharges confirm the analogy. Occasionally the adhesive form will extend to the larger trunks.

(2). *Suppurative Uterine Phlebitis, producing Puerperal Fever.*—The resemblance of the symptoms in these cases to those produced by injecting putrid matters into the blood, induced Dance to attribute them to the admixture of pus with the blood; but many cases of puerperal fever occur in which no traces of pus are found in the veins; and Tonnellé relates others in which no lesion in the uterus or its vessels was discoverable. Nonat, in the cases at the Hôtel Dieu, could discover no pus in the veins, but the lymphatics were filled with it. Our author states that, as puerperal fever usually arises amid circumstances favourable to the decomposition of animal matter, it is reasonable to believe that the fluids secreted by the veins in these cases undergo a decomposition, and thus infect the blood with which they are in contact.

(3). *Phlegmasia Dolens.*—This is now generally admitted to depend upon phlebitis. We need not detail symptoms of an affection so well known, but will merely quote the remark, that, even when the affection extends to the vena cava, it is not necessarily fatal, (and indeed it seldom is ever so from its mere local effects); for, supposing all inflammatory symptoms to have subsided, and there is simply an obstacle to the free passage of the blood, the collateral circulation will prevent any dangerous effects arising therefrom. M. Hourmann relates a case in point: a phlebitis of the vena cava inferior was produced by a fall, and was accompanied by swelling of the extremities, and difficulty of respiration. The superficial veins of the abdomen acquired a largely increased development, some of them equalling a large quill in diameter, and the patient soon manifested every sign of good health.

(4). *Capillary Phlebitis.*—M. Cruveilhier states, that in all phlegmasia the venous radicles are specifically affected; that, in fact, these parts are the essential seat of inflammation, *which is neither more nor less than a capillary phlebitis.* His reasons for this opinion may be found detailed in the 12th vol. of the Dict. de Med. and de Chir. Prat.; and we will only observe that among these is the fact, that injections of irritating substances into the arterial system only produced gangrene, while the same, thrown into the veins, gave rise to the production of phenomena of inflammation, among which were œdema of the limbs, apoplectic effusions into the substance of the muscles and cellular tissue, and abscesses around the suppurating veins.

2. *Varix.*—M. Briquet has endeavoured to prove that the varicose dilatation of the veins is not a mere distension of their walls, but results from "an actual increase of the number of molecules necessary for their nutrition." The varices of the genital organs were formerly divided into two varieties, viz. those of the spermatic chord termed *cirsocele,* and those of the testes termed varicocele; but the distinction is not now continued, both being considered but as different degrees of the same affection. Although, according to Delpech, varicocele is usually observed among adults and the aged, yet, Landouzy found, out of 45 examples, 13 were from 9 to 15 years old; 29 from 15 to 25; and 3 from 25 to 35. Females are occasionally subject to it. Of all veins those of the genital organs are most liable to become varicose, for reasons well stated by Morgagni in his 43d letter. He also explained that the affection is more frequently found on the left than on the right side, because the left spermatic vein does not discharge itself directly into the vena cava, but into the left emulgent vein. Callisen and Petit attribute this to the pressure of the fæcal matters in the sigmoid flexure of the colon. The veins are also usually more developed upon this side, while the testis is larger and hangs lower down. The affection is sometimes hereditary. There does not seem to be any relation between varicocele and a varicose state of the veins in other parts. Among 20 persons having varicose veins, Landouzy did not find one with a varicocele. When the varicocele becomes very large the testis may suffer from *atrophy,* and Landouzy says he has observed this nine times in 13 cases. Cooper and Velpeau regard this complication as exceedingly rare.

E. *The Treatment.*

1. *Phlebitis and its Consequences.*—An improved pathology has necessarily given rise to an improved treatment. Our attention is no longer turned to the relieving the nerve or tendon supposed to have been wounded in venesection. So after amputation we do not now seek to prevent the resorption of the pus by irritating applications, or by the use of blisters. We place the patient in as healthy a locality as possible, prevent the access of air to the wound, and moderate the inflammatory state of the veins by judicious antiphlogistics. As to puerperal fever, a chaotic mass of remedies has been employed: its treatment as a pure phlebitis has been too enthusiastically conducted. If it depends upon the decomposition of the fluids of the uterus, and their conveyance into the mass of the blood, as the author supposes, it is of the highest importance that the disposition to this may be checked, as far as possible, by permitting the patient to remain in a healthy atmosphere. At the Hôtel Dieu M. Caillard placed his puerperal patients in a ward, situated at the highest part of the house, isolated from the other patients, and taking care not to have it crowded. He lost but one patient in 25. In 1831 the ward by accident became very crowded, and numbers died of puerperal fever, while, as soon as it was restored to its original state, the mortality diminished. Every one knows the difference in puerperal mortality in hospital and private practice.

2. *Varix.*—We need not detail the various operations and modifications of operations proposed for this by Fricke, Velpeau, Delpech, Breschet, and others, as they are to be found in all recent surgical works; but we think the following observation well worthy of extract.

"It is the duty of the surgeon, in determining upon the operation, to be careful and not risk exposing his patient to consequences a hundred times more dangerous to him than the declining it altogether would entail. He must especially recollect this, as M. Velpeau remarks that it is very rare to obtain a radical cure of a varix. Generally, as soon as one venous trunk is obliterated, two or three neighbouring ones become varicose."

3. *Introduction of Air into the Veins.*—According to Amussat and Velpeau the preliminary pressure of the chest and walls of the abdomen is of no avail in preventing this occurrence; and the latter surgeon attaches no importance to the recommendation of Larrey and others to compress the vein between the heart and the wound, indeed it is frequently in a deep wound impossible to do so. Amussat thinks that, when the air has entered, suction by means of a glass syringe, introduced into the wounded vein, and directed towards the heart, should be employed.

### XI. On the Effect of a Moral Revulsion in the Treatment of Insanity. By *M. Leuret.*

M. Leuret believes that insanity will be little benefited by treatment directed to the condition of the bodily organs, unless there be any specific symptoms referible to their disordered state; and that our great dependence in treating nearly every case is in the judicious management of the moral treatment. We believe every body acknowledges the great importance of this now-a-days, and it seems no novelty upon the part of M. Leuret to announce that it may be conducted in two modes; either by endeavouring to restore the disordered functions to their normal condition, or by operating through the medium of those which remain unaffected, or indeed, as is usual, by a combination of the two. But it is especially upon the second mode, which he designates by the fanciful term *moral revulsion*, that the author relies.

"I have surrounded," says he, "many of my patients who were of an unsociable and insensible nature, with every means I believed calculated to awake their attention, and to arouse desires within their breasts. Other means failing, I have satisfied the cravings of some and created wants for others, in order to encrease the extent of their life of relation. For several, plays—for many, music—and for all, as frequently as possible, intercourse with intelligent persons, have been had recourse to. Some lunatics I have attacked as an adversary ready to struggle with their delusion to the utmost, opposing opposite passions to those which predominated, ever bearing in mind the precept of Esquirol, that to truly benefit a lunatic, one must love him and devote oneself to him; and in my most persevering struggles against his delusions, I have never forgotten that the unhappy creature with whom I was struggling was a human being. The insane must not be allowed to perceive that you are engaged in producing this revulsion or distraction of ideas, which they will soon do if care be not taken, and they then frequently thwart all your endeavours."

Pinel and Esquirol adopted this mode of proceeding, but those who believe insanity results from organic changes in the brain, consider moral treatment as a mere secondary matter. If, as the author states, he has an abundant supply of cases illustrative of the success of this plan, he has made a most unfortunate selection of the two he has published. In one of these a lady believed herself to be in turns a nun, a priest, and the pope, but was sufficiently sane in other respects to permit the author to engage her in various studies, in order to remove her delusion, of the existence of which she was perfectly cognizant, as also to promise him never to refer to it. In the other, a lady, who had remained obstinately dumb for eighteen months, was cured by her physician feigning dumbness, and by the fear of being obliged to submit to some disagreeable remedies.

Of such cures as these a very moderate experience in treating the insane might supply an abundance. In moral treatment, the author adds, little success, and often positive harm, has attended the attempts of directly attacking the cause of delusion. It is by revulsion that we may always, at least for the time, give new force to the understanding. The means of doing this are infinite, and the skill of the physician is shewn in their individual application—all that strikes the attention, arouses the energies, exercises the judgment, or excites the passions and desires, may be made available. The great difficulty to prevent, and yet the great object to overcome, is the state of languid torpor into which the insane are so apt to fall, even when surrounded by every means of distraction, when left to apply these of their own accord. They require continued care, indefatigable surveillance, and individual application of the means of revulsion. Bodily labour is useful, but it does not prevent the wandering of the ideas, in the same way in which well directed mental labour does—a labour which obliges them to concentrate their attention upon some determinate object. The greater numbers in which the insane are assembled together, the greater is the importance of a due exercise of their intellectual and moral powers. Schools for these objects should be established in every asylum; one has already been founded in the Bicetre, to which an instructor and music master have been appointed.

"Here, writing, reading, arithmetic, geography and history are taught: some patients are so occupied all day, and others only after employment in the fields and workshops. Some of the most heavy or stupified are exercised by dancing or gymnastics; while in the evening, all well enough are assembled together, and the day is terminated by music or singing."

No person can object to the moral treatment of insanity, and least of all any one in this country, where so much has been done of late in improving it—but we must suggest that there is a danger in carrying *intellectual labour*, so strongly recommended by the author, too far, for it may happen that in relieving one form of insanity we are laying the foundation for another.

By removing the delusions of the insane we do not always succeed in obtaining for them strong head-pieces.

### XII. A Memoir on the Anatomy of the Brain. By M. *Foville.*

This is a very important paper. The author states that his researches carry out in the brain the theory which Charles Bell has established respecting the spinal marrow, viz. the existence of different orders of roots of the spinal nerves, and of the bundles of the spinal marrow whence they arise. At first sight, the difficulty attending the investigation as regards the spine would seem to be encreased when it became extended to the brain; but in this latter we are at least certain that the optic and olfactory nerves are exclusively sensorial, in spite of all that has been of late said concerning the influence exerted by the fifth pair upon the organs these supply.

"If, then, I can demonstrate that there exists between the optic and olfactory nerves and the brain, cords and planes of nervous matter, combined with this organ in regions, perfectly distinct from those fibres, which, proceeding from the anterior pyramids, spread into the cortical substance of the convolutions, I shall have shewn the anatomical disposition of that, which pathology has already taught us, viz. the distinct routes by the medium of which the brain influences the muscles, and those by which the impressions derived from the organs of the senses reach it."

To give an abridgement of this demonstration would render it unintelligible, so that, not having sufficient space at command, we must defer presenting the entire translation until another opportunity. We can only glance at some of the author's conclusions.

"Thus, the corpus callosum is a large commissure of the encephalic prolongations of the posterior bundles of the spinal marrow, and the plane of the hemisphere is the uninterrupted prolongation of the anterior bundles of the spinal marrow. * * * * * * *

The anterior commissure unites, from one side to another of the brain, those parts which are at once continuous with the posterior bundles of the spinal marrow, and with the sensorial nerves of the brain. The auditory nerve also, although arising in so remote a part of the brain, may be traced into those parts of the organ we have been describing. * * * *

On the other hand, the fibrous planes of the hemispheres, emanating from the anterior pyramid of the spinal marrow, proceed to the internal surface of the cortical substance, without any communication by means of commissures uniting to fibrous planes of the one hemisphere with those of the other. Moreover the order of parts spring from the posterior portion of the spinal marrow, present in their course greyish swellings which most anatomists regard as ganglions, with which they are in connection, and from which they receive numerous fibres: but the other order of parts traversing the circle formed by the first and their ganglions, are never brought into direct relation with these ganglions.

I draw the following physiological corollaries:—

1. The fibrous parts of the brain seem to be simple conductors, the cortical substance of the convolutions in which these terminate being the essentially active portion. 2. The fibrous portions, intermediate between the convolutions and the anterior pyramids, are devoted to the transmission of the influence which determines voluntary movements. 3. The fibrous portions, communicating between the sensorial nerves and the cortical substance, and in whose course ganglionic swellings are found, transmit the sensorial impressions to the brain.

It is lesion of the cortical substance that is usually observed in changes of the brain produced by insanity. The atrophy of the convolutions, and the substance of the brain, so frequently seen in dementia, seems to result from the abolition of the functions of the grey portion—the fibrous substance in connexion with it thence becoming atrophied, as do the optic nerves in the blind. Pathological anatomy presents many examples of lesions of the portions of the brain intermediate between the convolutions and anterior pyramids, of which the cross paralysis of the active organs of motion is a frequent consequence. The fibrous substance intermediate between the convolutions and the organs of the senses is less frequently affected pathologically:—the existence of so many commissures render the abolition of the transmission of the impressions difficult to effect, for, by means of these, each sensorial nerve is in relation with each hemisphere, while each motor nerve is connected with but one.

Comparative anatomy teaches us that in brutes the intermediate portions between the senses and the brain, become developed in proportion to the activity of their organs of sight and smell; while those parts, the lesions whereof in man are attended with intellectual disorder, or muscular paralysis, are found to be in animals proportionally feeble to their slight manifestation of the functions supposed to be dependent upon them." 682.

The author terminates his paper by observations on the relative form of the cranium to that of the brain; and endeavours to prove that the various frontal, occipital, and parietal protuberances depend upon the formation of the ventricle.

"The form of the brain and the form of the cranium are alike determined by the form of the proper serous sacs of the hemispheres, constantly filled with their natural fluid."

### XIII. Anthelmintic Substances employed in Abyssinia. By M. *Aubert*, M.D.

M. Aubert having resided some time in Abyssinia, observed that the inhabitants suffered but from two diseases, viz. *syphilis*, which has ravaged the country for 300 years, and for which no remedy is known; and *tænia*, which prevails almost universally, but for which they have three most excellent remedies. These are the flowers of the *coussou*, a magnificent and lofty tree, the bulb of the *abbatsjogo*, and the bark of the *bisenna*. These plants he thinks might be naturalized in Europe. He attributes the almost universal prevalence of tænia to the circumstance of the Abyssinians living upon *raw meat*. The few Mussulmen, who are in the country, abstaining from this, never become affected, and those Europeans, who lived as the Abyssinians, became like them, infested with the worms, while those who avoided doing so escaped.

### XIV. Reduction of a Congenital Dislocation of the Humerus at the end of Sixteen Years. By Dr. *Gaillard*.

The head of the bone was thrown backward into the infra-spinal fossa of the scapula. The reduction was accomplished very gradually, by means of the pulleys, at several sittings, of about 20 minutes each, and did not occasion much pain. Great difficulty was experienced in maintaining the bone in its place, as every muscular movement threw it out again, when however it was easily reduced again. Considerable pain and irritation followed the attempt to maintain the parts in situ by position and bandages.

The cure occupied several months by reason of the pain and swelling, but was eventually complete ; and the author, seeing the patient two years after, found the head of the humerus maintained in its proper situation, and that all the normal movements of the extremity could be executed.

Our readers will have observed that this volume of the Transactions of the Academy contains much valuable matter.

